# Preclinical evaluation of radiation therapy of *BRCA1*-associated mammary tumors using a mouse model

**DOI:** 10.7150/ijbs.53667

**Published:** 2021-01-31

**Authors:** Eun Ju Cho, Jong Kwang Kim, Hye Jung Baek, Sun Eui Kim, Eun Jung Park, Bum Kyu Choi, Tae Hyun Kim, Dong Hoon Shin, Young Kyung Lim, Chu-Xia Deng, Sang Soo Kim

**Affiliations:** 1 Research Institute, National Cancer Center, Goyang, 10408, Korea.; 2 Proton Therapy Center, National Cancer Center Hospital, Goyang, 10408, Korea.; 3 Cancer Centre, Faculty of Health Sciences, University of Macau, Macau SAR 999078, China.

**Keywords:** BRCA1, irradiation, AZD2281, precision medicine

## Abstract

Although germline mutations in *BRCA1* highly predispose women towards breast and ovarian cancer, few substantial improvements in preventing or treating such cancers have been made. Importantly, BRCA1 function is closely associated with DNA damage repair, which is required for genetic stability. Here, we examined the efficacy of radiotherapy, assessing the accumulation of genetic instabilities, in the treatment of *BRCA1*-associated breast cancer using a *Brca1*-mutant mouse model. Treatment of *Brca1*-mutant tumor-engrafted mice with X-rays reduced tumor progression by 27.9% compared with untreated controls. A correlation analysis of irradiation responses and biomarker profiles in tumors at baseline identified differences between responders and non-responders at the protein level (pERα, pCHK2, p53, and EpCAM) and at the SOX2 target expression level. We further demonstrated that combined treatment of *Brca1*-mutant mammary tumors with irradiation and AZD2281, which inhibits PARP, significantly reduced tumor progression and extended survival. Our findings enhance the understanding of DNA damage and biomarker responses in *BRCA1*-associated mammary tumors and provide preclinical evidence that radiotherapy with synthetic DNA damage is a potential strategy for the therapeutic management of *BRCA1*-associated breast cancer.

## Introduction

BRCA1 (breast cancer 1, early onset) is a tumor-suppressor protein that plays a critical role in maintaining genomic integrity through regulation of important cellular processes, including genetic stability, DNA damage repair, centrosome duplication, apoptosis, and cell-cycle control 1-3. Germline mutations in *BRCA1* are responsible for a considerable proportion of hereditary breast and ovarian cancers 4. Women with germline mutations in *BRCA1* have a 57% (95% confidence interval [CI], 47%-66%) risk of developing breast cancer and a 40% (95% CI, 35%-46%) risk of developing ovarian cancer by the age of 70 5. Gene and protein expression profiling has revealed that cancers arising as a result of a BRCA1 deficiency show triple-negative and basal-like properties, tend to be aggressive, and typically have a poor prognosis 6,7. The National Comprehensive Cancer Network (NCCN) recommends that *BRCA1* mutation-positive women undergo periodic breast screening and consider mastectomy and salpingo-oophorectomy to reduce their cancer risk. When tumors occur, the recommended treatment option has been resection of tumors followed by adjuvant chemotherapy (NCCN guideline Ver. 2.2017). However, resection and chemotherapy may not be applicable and effective for all patients. Thus, given the numerous hurdles encountered by efforts to develop and validate suitable therapies in clinical trials, a means for improving the treatment of *BRCA1*-associated breast cancer is urgently needed.

Recently FDA approved poly-(ADP-ribose) polymerase (PARP) inhibitor therapy in recurrent or metastatic breast cancer harboring germline *BRCA1/2* mutations. While FDA guidelines indicate olaparib (AZD2281) and talazoparib for HER2-negative disease, the panel supports their use in any breast cancer subtype associated with germline *BRCA1* or *BRCA2* mutations 8,9 (NCCN guideline Ver. 2.2020). Treatment of *Brca1*-mutated tumor-bearing mice with olaparib was found to induce synthetic lethality by disrupting homologous recombination and inhibiting other repair pathways 10,11,12,13. A subsequent clinical trial showed that progression-free survival at a median follow-up of 14 months was 2.8 months longer and the risk for disease progression or death was 42% lower with olaparib monotherapy compared with conventional chemotherapy 9. Accordingly, PARP inhibition is currently considered a targeted therapy for *BRCA1*-associated breast cancer.

Radiotherapy, which delivers high-energy particles or waves to destroy or damage cancer cells, is an effective treatment for the majority of localized solid cancer types. Radiation exerts its effects by making small breaks in the DNA inside cancer cells, thereby preventing cells from growing and ultimately causing them to die 14. Unlike chemotherapy and other treatments that expose the whole body to cancer-fighting drugs, radiation therapy is a local treatment directed at and affecting only the part of the body needing treatment and is suitable treatment for breast cancer at almost every stage 15. In *BRCA1*-associated breast cancer, irradiation-induced DNA breakage increases the lethality against *BRCA1*-mutant tumor cells, while allowing surrounding normal tissue to survive by virtue of continued appropriate DNA repair activity 16. Whereas, it is also possible that irradiated *BRCA1*-mutnat tumor cells are liable to having mutations for insufficiency of DNA damage repair. Several attempts were performed whether BRCA1/2 mutation carriers with radiotherapy display higher incidence of secondary malignancy comparing BRCA1/2 mutation carriers with mastectomy or sporadic breast cancer patients. However, these studies could not find a significant association between BRCA1/2 mutation carriers with radiotherapy and the secondary risk of tumor including contralateral breast cancer 17 18. To assess the application of radiotherapy to *BRCA1*-associated breast cancer, we examined the benefit of radiation to suppress the progression of *Brca1*-mutant tumors from *Brca1^co/co^MMTV-Cre* mice, which develop tumors that mimic human *BRCA1* mutation-related mammary tumors. We also evaluated the effect of a combination regimen of radiotherapy and the PARP inhibitor AZD2281 on tumor progression *in vivo*.

## Materials and methods

### Animal experiments

*Brca1* exon 11-deleted conditional-knockout (*Brca1-co*), and *MMTV-Cre* transgenic mice were provided by the National Cancer Institute (NCI, USA) mouse repository. All procedures involving animals and their care were approved by the Institutional Animal Care and Use Committee of the National Cancer Center (Goyang, Korea). Female *Brca1*-mutant mice were generated by intercrossing *Brca1* conditional-knockout mice, and *MMTV-Cre* mice, originally generated by Dr. Deng, and Dr. Hennighausen, respectively 19, 20. Mice carrying mutant alleles were identified previously described 21. After 8 months of age, mice were examined weekly for the occurrence of tumors. For tumor allografts, spontaneously formed primary tumors obtained from *Brca1^co/co^MMTV-Cre* mice were orthotopically implanted into 5-week-old female BALB/cOlaHsd-*Foxn1^nu^* (*Balb/c-nu*) mice (Orient-Harlan Laboratories, Sungnam, Korea). After each grafted tumor reached a volume of ~1,000 mm^3^, the tumor tissue was excised, trimmed with a tissue slicer, and heterotopically reimplanted into thigh of recipient mice. After implantation, the recipient mice were left untreated or were treated with X-ray radiation, AZD2281, or combination, as indicated. To examine the progress of tumorigenesis, we monitored the mice three times a week from the initial treatment using calipers. Tumor volume (in mm^3^) was calculated according to the following formula: V = 0.5 × d^2^ × D, where d is the shorter diameter and D is the longer diameter. Tumor growth was assessed as the ratio of the tumor volume (RTV) at a given time to that recorded at the initiation of treatment (baseline tumor); assessments were made until the tumor volume reached ~3,000 mm^3^.

X-ray was obtained using a 225 kV accelerator (XenX; Xstrahl, Camberley, England). The radiation dose was delivered to the tumors at a 30-cm source-surface distance, with a 1 cm circular collimated field size and a dose rate of 2.5 Gy/min. At each point, tumored female mice were immobilized in a special stage with anesthesia and irradiated. AZD2281 was purchased from Abmole Bioscience (Houston, TX, USA), prepared as described previously 21. In the animal experiments, any serious adverse event was not detected including dramatic loss of body weight.

### Cell culture

MCF7 cells were obtained from the American Type Culture Collection (Manassas, VA, USA). The authenticity of human cell lines was confirmed by short tandem repeat (STR) analysis performed by the genomics core of the National Cancer Center. *Brca1^Δ11/Δ11^Tp53^+/-^* and *Brca1^Δ11/Δ11^53BP1^-/-^* mouse mammary tumor cell lines were generated from the corresponding tumors as described previously, and were altered p53 and 53BP1 in addition to BRCA1, respectively 22,23. For growth assays, cells were plated at 5 × 10^3^ cells per well in 4-well plates in quadruplicate, with or without the indicated treatments, and cell viability was determined using an *In vitro* Toxicology Assay Kit (Sigma, St. Louis, MO, USA) according to the manufacturer's instructions. *BRCA1* expression in MCF7 cells was knocked down by transfecting cells with a pool of three *BRCA1*-targeting small interfering RNAs (siRNAs; Santa Cruz, Dallas, TX, USA) or scrambled siRNA controls (Dharmacon, Lafayette, CO, USA) using Lipofectamine 2000 (Invitrogen, Waltham, MA, USA) according to the manufacturer's protocol.

### Histology and immunodetection

For histology, tissues were fixed in 10% (v/v) formalin, embedded in paraffin, sectioned, stained with hematoxylin and eosin (H&E), and examined by light microscopy. After evaluation of H&E tissue sections of each case, representative neoplastic areas were marked, and the corresponding paraffin block was retrieved. A tissue core 3.0 mm in diameter was obtained from each selected block using Quick-Ray manual tissue microarray system (UNITMA, Seoul, Korea). Tissue microarrays contained 30 tissue cores of multiple control and treated tissues were generated and compared the expression of biomarkers. Immunoreactive proteins were detected using indicated primary antibodies and Zymed Histostain kit (Invitrogen, Waltham, MA, USA) according to the manufacturer's instructions. The following antibodies were used in IHC staining: anti-cleaved Caspase 3 (Asp175), anti-EpCAM, anti-F4/80, anti-phospho-p53 (Ser15), anti-phospho-Rb (Ser807/811) (all from Cell Signaling Technology, Danvers, MA, USA); anti-phospho-Histone H3 (Ser10) (Millipore, Temecula, CA, USA); and anti-PCNA (Atlas Antibodies, Bromma, Sweden); anti-p53 (Novocastra, Newcastle upon Tyne, UK).

Western blot analysis was carried out according to standard procedures using enhanced-chemiluminescence detection (GE Life Science, Chicago, IL, USA). Tumor tissue lysates were prepared using an electric homogenizer for 30 seconds after the addition of lysis buffer. The following antibodies were used: anti-β-Actin, anti-phospho-AKT (Ser473), anti-phospho-ATM (Ser1981), anti-phospho-ATR (Ser428), anti-Caspase 3, anti-cleaved Caspase 3 (Asp175), anti-Caspase 9, anti-cleaved Caspase 9 (Asp330), anti-β-Catenin, anti-phospho-CHK2 (Thr68), anti-phospho-ERα (Ser118), anti-GAPDH, anti-phospho-MAPK (Thr202/Tyr204), anti-p21, anti-PARP, anti-phospho-S6 (Ser235/236), anti-phospho-mTOR (Ser2448), anti-phospho-Rb Ser807/811) (all from Cell Signaling Technology, Danvers, MA, USA); anti-β-Actin, anti-Bcl2 anti-BRCA1, anti-Cyclin D1, anti-p53 (all from Santa Cruz, Dallas, TX, USA); and anti-Ki-67, anti-p53 (Novocastra, Newcastle upon Tyne, UK); anti-LC3B (Novus, Centennial, CO, USA); anti-PCNA (Atlas Antibodies, Bromma, Sweden). Horseradish peroxidase-conjugated goat anti-rabbit or anti-mouse antibodies (Jackson Immuno Research, West Grove, PA, USA) were used as secondary antibodies as appropriate.

### Omics data analysis

Raw data obtained from a set of 12 samples were normalized using Cufflinks RNAseq workflow 24. Spearman's rank correlation coefficients (*P* < 0.05) were calculated for each gene to identify genes that were correlated with response to irradiation or drug treatment. The highly correlated genes (HCG) that overlapped with known hallmark genes were selected as markers, and a heat map was generated using the z-scores of their normalized expression in fragments per kilobase per million mapped fragments (FPKM) as input to the heat map function of the Superheat R open source package. Samples were sorted to show a good correlation between the ratio of tumor volume (RTV) and gene expression patterns.

All known concepts of gene sets with our markers were analyzed using MSigDB, a database of known hallmark gene sets, one of the most widely used and comprehensive databases for performing gene set enrichment analysis (https://www.gsea-msigdb.org). Over 10,000 gene sets including expert-curated hallmark genes were used for making enrichment map. The node cutoff *p*-value of 0.01 and edge cutoff score of 0.5 (similarity) were used for mapping an integrated network of enriched gene sets. Nodes in the network were colored according to enriched categories and node size is proportional to enrichment significance.

For survival analysis, patient survival data and normalized mRNA expression data (Illumina HiSeq Ver.2) of breast cancer (BRCA) were downloaded from The Cancer Genome Atlas (TCGA) from the NCI, following TCGA Human Subject Protection and Data Access Policies. We selected those patient samples with radiation treatment history and used them for downstream analysis.

## Results

### Loss of BRCA1 enhances sensitivity to irradiation

DNA damage induces alterations in BRCA1, causing formation of discrete nuclear foci, co-localization with Rad51 and dose-dependent phosphorylation, among other effects 25. In addition, loss of BRCA1 leads to hypersensitivity to DNA-damaging treatments, indicating that BRCA1 is required for a proper DNA-damage response 26. Indeed, cells and mice with a loss of BRCA1 exhibit abnormalities in DNA, suggesting that BRCA1 deficiencies are associated with genetic instabilities that eventually lead to tumorigenesis 19,22.

**Figure 1:**
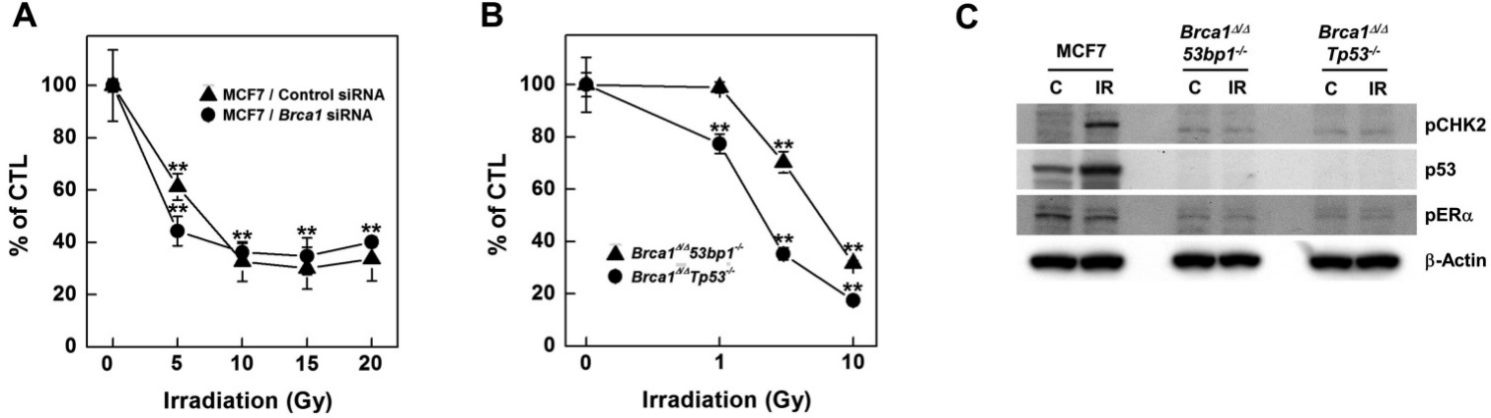
** Irradiation reduces the survival of *BRCA1*-down-regulated and -mutated tumor cells.** (A) MCF7 cells were transfected with control or *BRCA1* siRNA, and then treated with the indicated dose of irradiation. Irradiation-induced survival was estimated using MTT assays. (B) The survival of *Brca1*^Δ^*^11/^*^Δ^*^11^53bp1^-/-^* (triangle) and *Brca1*^Δ^*^11/^*^Δ^*^11^Tp53^-/-^* (circle) mammary tumor cell lines was estimated in the presence of the indicated dose of irradiation. Each number represents survival relative to that in the absence of irradiation (**P < 0.01). (C) MCF7, *Brca1*^Δ^*^11/^*^Δ^*^11^53bp1^-/-^* and *Brca1*^Δ^*^11/^*^Δ^*^11^Tp53^-/-^* mammary tumor cells were exposed to irradiation (10 Gy) and their protein expression patterns were analyzed by Western blotting. β-Actin was detected as a loading control.

To investigate radiation effects on BRCA1-deficient cells, we knocked down *BRCA1* expression in MCF7 breast cancer cells using small interfering RNA (siRNA) and assessed survival of the resulting *BRCA1*-knockdown cells using MTT [3-(4,5-dimethylthiazol-2-yl)-2,5-diphenyltetrazolium bromide] assays following exposure to increasing doses of irradiation (Fig. 1A). Radiation doses greater than 5 Gy significantly reduced the survival of *BRCA1*-knockdown MCF7 cells; similar results were obtained in control siRNA-transfected MCF7 cells. However, at a dose of 5 Gy irradiation, survival of *BRCA1*-siRNA-transfected MCF7 cells (44%) was reduced compared with that for control siRNA-transfected cells (61%), suggesting that alterations in BRCA1 tend to promote hypersensitivity to low doses of irradiation. We also analyzed survival of mammary tumor cell lines harboring *Brca1* mutants following exposure to radiation, demonstrating that these cells exhibited radiation hypersensitivity similar to that of MCF7 cells (Fig. 1B). These *Brca1*-mutant tumor cells, showing heightened responsiveness to radiation, also displayed altered expression patterns of certain proteins, including phospho-CHK2 and p53 (Fig. 1C).

**Figure 2:**
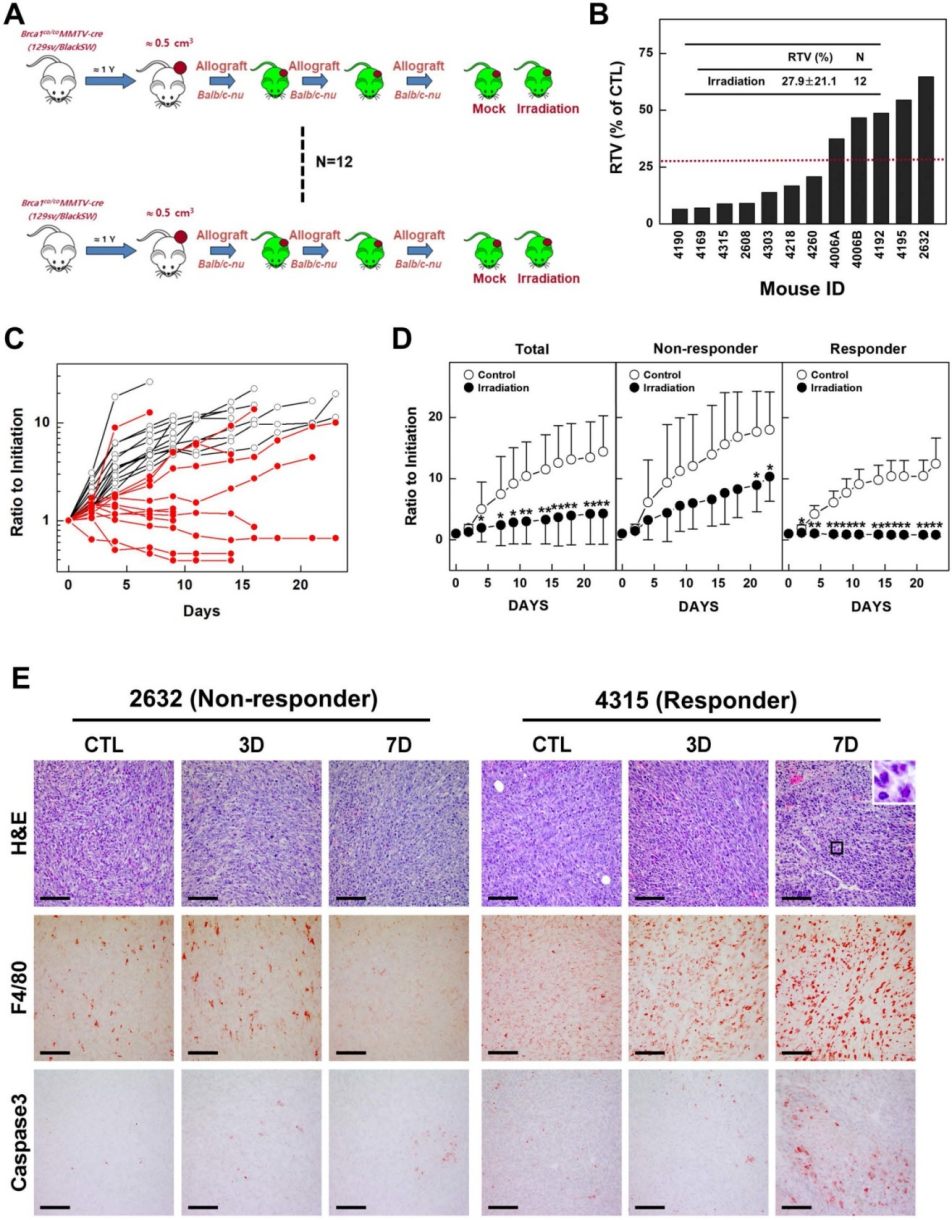
** Therapeutic effects of irradiation in a BRCA1-deficient tumor transplantation model.** (A) Overview of the allograft model and radiotherapeutics. Twelve spontaneously developed mammary tumors were collected from *Brca1^co/co^MMTV-Cre* mice and transplanted into *Balb/c-*nude mice. Growth of the corresponding tumors in sham-treated mice versus mice treated with irradiation (20 Gy) is shown. When the tumor of any mouse implanted with the same original tumor reached ~3,000 mm^3^, control and treated mice implanted with the same tumor were sacrificed and examined. (B) Graph shows calculated RTVs (RTV of treated tumor/RTV of control tumor × 100) for tumors at 1 week after irradiation. (C) Responses of allograft *Brca1*-mutant mammary tumors to irradiation. Graphs show RTVs of control (black line) and treated (red line) mice post-treatment relative to baseline (start of treatment). (D) Responses of *Brca1*-allograft tumors to irradiation, segregated based on RTV (non-responder, RTV > 27.9; responder, RTV < 27.9). Numbers represent means ± SD (*P < 0.05, **P < 0.01). (E) Histological analyses of irradiated tumors from non-responder and responder mice at the indicated days after irradiation are shown. Inset (upper right) in H&E-stained images of responder mouse 4315 on day 7 is a magnification of the boxed area showing multinucleated gigantic cells following irradiation. Scale bars: 100 µm.

Previous studies have reported that mammary tumors spontaneously generated in *Brca1*-mutant mice can be orthotopically transplanted into female mice without losing their original phenotype, gene expression profile, or sensitivity to anticancer agents 10,27. To examine whether radiotherapy could effectively suppress *BRCA1*-associated breast cancer, we tested the efficacy of X-ray radiation in an *in vivo* allograft model. To this end, we collected 12 tumor samples from spontaneously developed mammary tumors in *Brca1^co/co^MMTV-Cre* mice and transplanted them into the hind legs of *Balb/c*-nude female mice. We then used this model to test the efficacy of radiotherapy by comparing treated and non-treated tumors with the same origin (Fig. 2A). After tumors reached a size of ~0.5 cm^3^, we applied X-ray radiation at a dose of 20 Gy using a 1-cm circular collimated field size to protect the rest of the body. One week after irradiation, the overall relative tumor volume (RTVs; treated vs. non-treated) for mice bearing *Brca1*-mutant tumor allografts was 27.9% for mice treated with X-ray irradiation compared with those left untreated (Fig. 2B). A comparison of baseline and progressed tumor volumes in treated (Fig. 2C, red line) and non-treated (Fig. 2C, black line) *Brca1*-mutant tumor-bearing mice showed that mammary tumor volumes increased 2.39-fold after irradiation and more than 7 times in the absence of treatment. Interestingly, some mice showed a rapid reduction in tumor volume after treatment, whereas in some cases responses with and without treatment were not distinguishable. An analysis of tumors in individual mice treated with X-rays revealed that 7 of 12 mice exhibited a reduction in tumor volume in response to irradiation greater than the average of 27.9%, whereas the reduction was less than average in the remaining 5 mice. Further analyses showed that non-responder mice (n = 5) showed 4.41-fold increment after irradiation, which translates to only a 47.3% compared with non-treated mice (9.32-fold increment), whereas responders showed a 6% decrease in tumor volume after irradiation compared with non-treated mice (6.12-fold increment) (Fig. 2D and Table 1). In addition, histological analyses revealed that irradiated tumors from responders exhibited multinucleated giant cells with increased numbers of macrophages and apoptosis markers, including F4/80 and cleaved Caspase 3, compared with tumor tissues from non-treated and irradiated non-responder mice (Fig. 2E). These findings suggest that radiation exposure attenuates the growth of *Brca1*-mutant tumors and is more effective in radiation-sensitive individuals.

### Analysis of irradiation response-associated biomarkers

**Figure 3:**
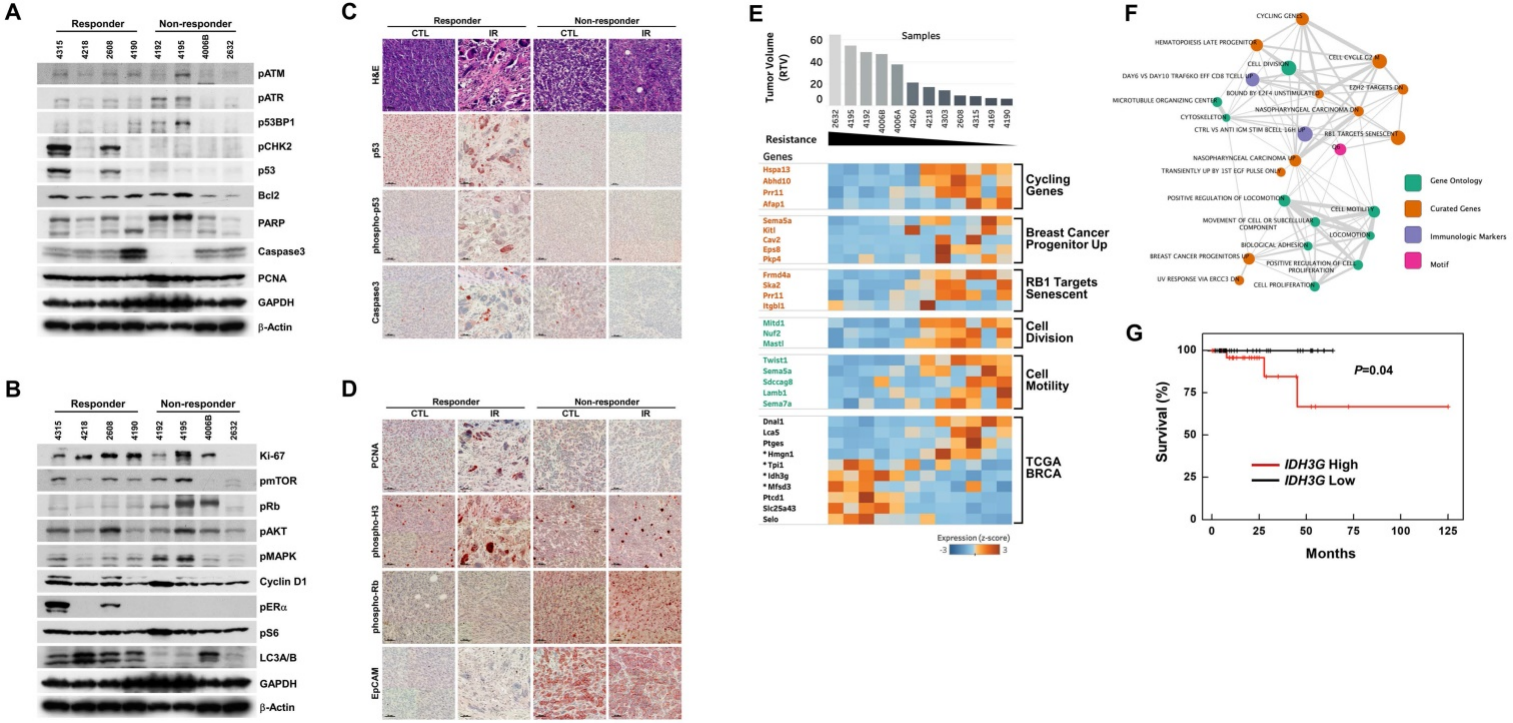
**Analysis of irradiation response-associated biomarkers.** (A and B) Protein expression patterns of baseline tumors in irradiation-sensitive and -insensitive groups. GAPDH and β-Actin were used as loading controls. (C and D) Histological analyses of control and irradiated tumors in responders and non-responders are shown. Scale bars: 100 µm. (E) Heat map showing correlations of selected genes with responses to irradiation in the allograft model (rho > 0.6 or rho < -0.6, P < 0.05). Tumor samples were sorted with respect to their RTV to highlight correlations with gene expression. Genes marked with “*” were cross-validated in the TCGA breast cancer (BRCA) mRNA expression dataset of radiation therapy patients. (F) Integrated enrichment map of the selected genes using the MSigDB molecular signature database. (G) Analysis of survival based on expression of the *IDH3G* gene using TCGA BRCA expression data. Patients with high expression showed a worse survival rate.

Although the overall results of irradiation showed improvement in cohorts of mice harboring *Brca1*-mutant tumors, some individuals exhibited better response to this X-ray-induced synthetic DNA-damaging strategy. To increase the potential clinical efficacy of radiotherapeutics against *BRCA1*-associated breast cancer, it would be helpful to distinguish potential responders from non-responders before initiation of treatment. In an effort to identify candidate prognostic markers, we classified cases based on their responsiveness to irradiation, and further analyzed protein patterns in untreated baseline tumor tissue. Western blot analyses showed that the levels of phospho-ERα (Ser118), phospho-CHK2 (Thr68), and p53 were frequently increased in the responder group compared with the non-responder group (Fig. 3A and 3B). We and other investigators previously showed that estrogen signaling alters cell proliferation and expression of proteins responsible for DNA-damage repair, including BRCA1 and p53 28,29, indicating that *BRCA1*-associated tumors in which the ERα/CHK2/p53-dependent DNA-damage-response pathway is elevated are suitable candidates for radiation treatment. In addition, irradiated tumors of responders exhibited multinucleated giant cells and showed high levels of nuclear p53 by immunohistochemistry (Fig. 3C). In contrast, Western blotting and tissue immunostaining showed that levels of phospho-Rb (Ser807/811) were frequently high in non-responders, which also displayed intense EpCAM staining in tumor cells that was not altered after irradiation (Fig. 3D).

As an alternative strategy, we examined gene expression patterns in the corresponding baseline tumor tissue samples according to their responsiveness to X-ray treatment. Accordingly, we collected 12 non-treated allograft mammary tumors and screened their entire transcriptomes using mRNA sequencing and the Cufflinks computational pipeline to predict which genes were associated with responsiveness following irradiation of *Brca1*-deleted tumors. We identified 158 genes whose expression correlated with RTV (correlation > 0.6 or < -0.6, *P*-value < 0.05) (Fig. 3E, and Supplementary Table 1). To assess potential consequences of changes in the 158 putative markers following radiation treatment, we identified downstream pathways of these markers that might specifically affect the regulation of radio-resistance or -sensitivity. To this end, we constructed an enrichment map of statistically over-represented pathways and performed a Gene Ontology (GO) analysis of these 158 genes using the Molecular Signatures Database (MSigDB) 30. This analysis showed that 52 genes were connected to various pathways and experimental signature genes curated from the literature (Fig. 3F, and Supplementary Table 2). In particular, it revealed several clusters of genes associated with cell motility and cell division (*P*-value < 0.01). Notably, one cluster was associated with cancer stem cell markers up-regulated in “Breast cancer progenitors” (*P-*value = 0.005). The lack of these cancer stem cell markers, specifically *SEMA5A* (semaphorin 5A), *KITL* (KIT ligand, stem cell factor), *CAV2* (caveolin 2), *EPS8* (epidermal growth factor receptor pathway substrate 8) and *PKP4* (plakophilin 4), was associated with acquired resistance to radiation in this study (Fig. 3E). Another cluster of “RB1 Target senescent” genes might also be associated with resistance to radiation through dysregulation of genes involved in DNA replication targeted by the tumor suppressor RB1 31. GO analyses also revealed that 35 of the genes in the constructed map were involved in biological adhesion (GO:0022610), cellular proliferation (GO:0008283), cytoskeleton (GO:0005856), locomotion (GO:0040011), movement of cell or subcellular component (GO:0006928), or protein modification processes (GO:0036211).

We next selected four genes from among the identified radiation-response marker genes in our mouse model that cause survival differences between patients with high and low expression status using a TCGA breast cancer (BRCA) patient cohort with a radiation treatment history (Fig. 3E). For instance, *IDH3G* (isocitrate dehydrogenase 3 non-catalytic subunit gamma) was activated in the radiation-non-responder group in our study and in the TCGA BRCA cohort, where it was associated with poor survival, consistent with the results of our study (*P*-value = 0.04, log-rank test) (Fig. 3G). The other three cross-validated genes, *HMGN1* (high-mobility group nucleosome binding domain 1), *TPI1* (triosephosphate isomerase 1) and *MFSD3* (major facilitator superfamily domain containing 3), could also be good candidate markers for predicting radiation-responsiveness in patients before treatment, but will require further validation studies (Fig. 3E).

### Combined effects of radiation and PARP inhibition

**Figure 4:**
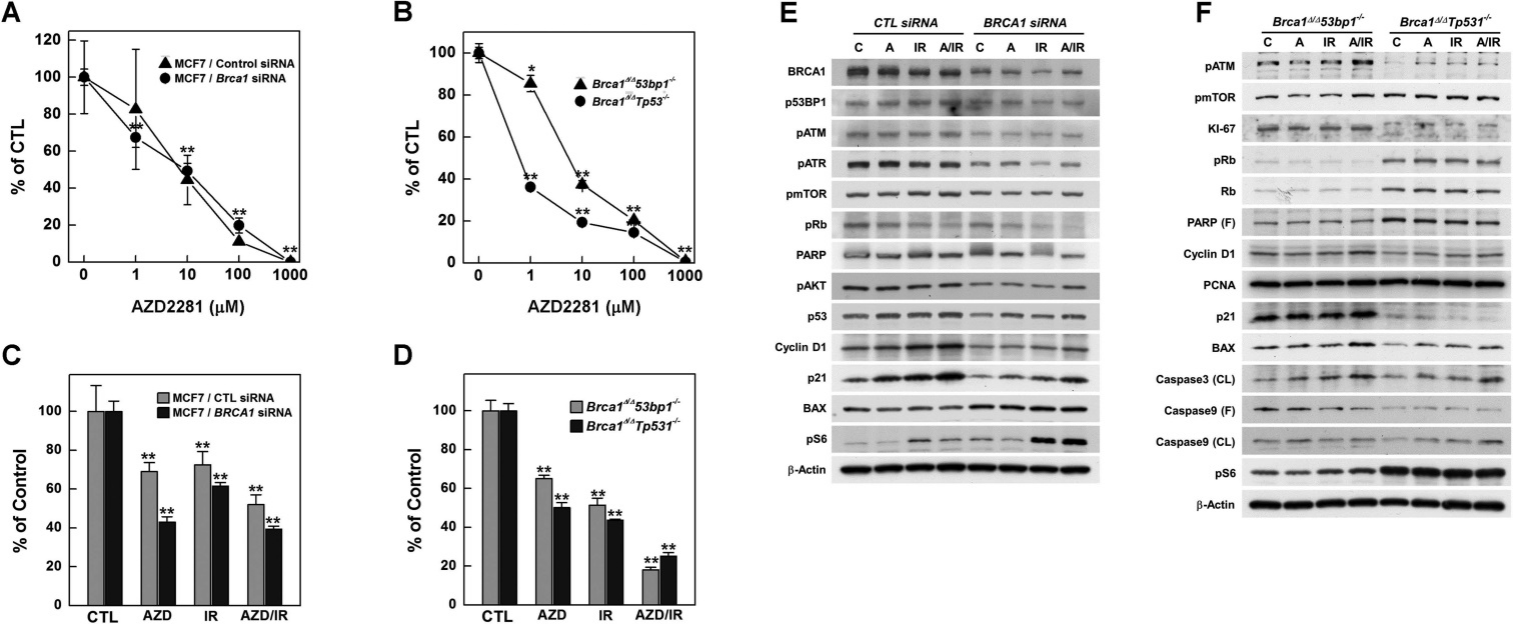
** Combination of PARP inhibition and irradiation reduces the survival of BRCA1-altered tumor cells.** Control or *BRCA1* siRNA-transfected MCF7 cells (A) and mouse *Brca1*-mutant cells (B) were treated with increasing concentrations of AZD2281 in the presence of 3 Gy irradiation and their survival was measured by MTT assay. (C) Survival of MCF7 cells was estimated by MTT assay after treatment with AZD2281 (10 μM), irradiation (3 Gy), or their combination. (D) MTT assays of mouse *Brca1*-mutant cells after treatment with AZD2281 (*Brca1*^Δ^*^11/^*^Δ^*^11^53bp1^-/-^*; 3 μM, *Brca1*^Δ^*^11/^*^Δ^*^11^Tp53^-/-^*; 0.3 μM), irradiation (3 Gy), or their combination. The numbers indicate relative survival compared with untreated (vehicle-exposed) controls. Numbers represent means ± SD (*P < 0.05, **P < 0.01). *BRCA1* siRNA-transfected MCF7 cells (E) and *Brca1*^Δ^*^11/^*^Δ^*^11^53bp1^-/-^* and *Brca1*^Δ^*^11/^*^Δ^*^11^Tp53^-/-^* mammary tumor cells (F) were treated as above and their protein expression patterns were analyzed by Western blotting. β-Actin was detected as a loading control.

AZD2281 (Olaparib) is an inhibitor of PARP, which senses DNA breaks and plays essential roles in damage repair 32. Treatment of *Brca1*-mutated tumor-bearing mice with AZD2281 was found to inhibit tumor growth alone and to potentiate the clinical effectiveness of DNA-damaging anticancer agents when used in a combined treatment regimen 10,11. To determine whether the combination of irradiation and PARP inhibition is effective in suppressing BRCA1-deficient breast cancer, we first tested the efficacy of irradiation together with AZD2281 in MCF7 cells transfected with siRNA against* BRCA1*. The lethality of radiation (3 Gy) against *BRCA1*-knockdown cells was increased with increasing concentrations of AZD2281 (Fig. 4A). A similar pattern was also found for *Brca1*^Δ*11/*Δ*11*^*53bp1^-/-^* and *Brca1*^Δ*11/*Δ*11*^*Tp53^-/-^* mammary tumor cells (Fig. 4B). Notably, combined treatment with radiation and AZD2281 significantly reduced survival of tested mammary tumor cell lines (Fig. 4C and 4D). A further examination of expression patterns of proliferation-related proteins in *in vitro* co-treatment experiments failed to identify effectors in common (Fig. 4E and 4F).

**Figure 5:**
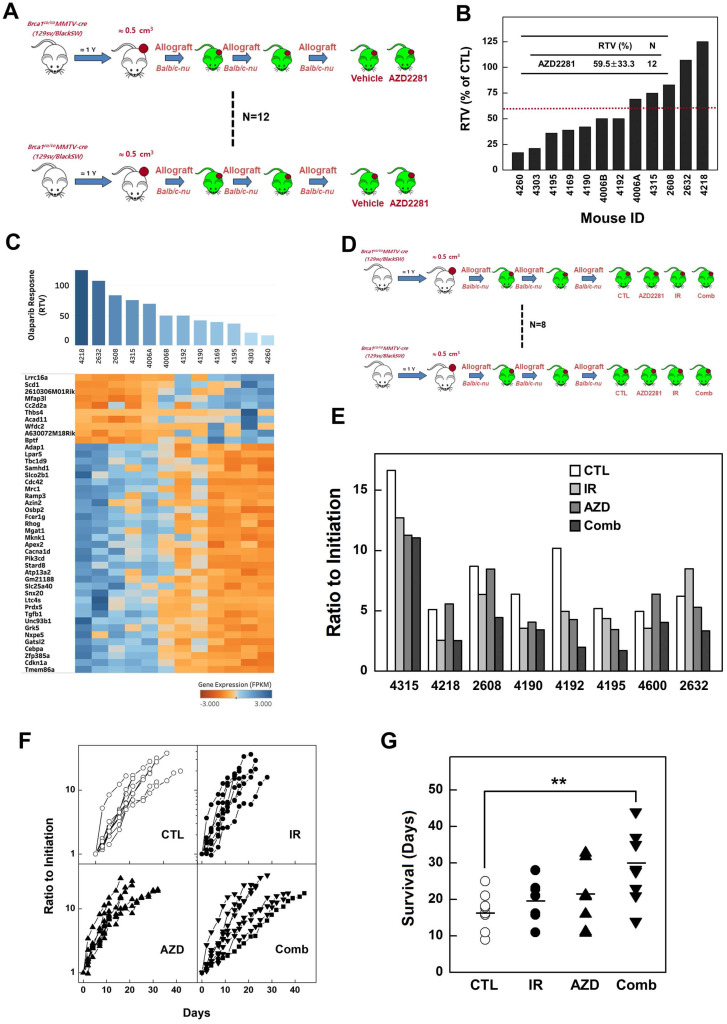
** Combined treatment with irradiation and AZD2281 suppresses the growth of *Brca1*-mutant breast tumors.** (A) Overview of the transplanted mouse model for testing PARP inhibition. Twelve sets of mammary tumors from *Brca1^co/co^MMTV-Cre* mice were orthotopically transplanted into *Balb/c-*nude mice. Growth of the corresponding tumors in sham-treated mice versus mice treated with AZD2281 (100 mg/kg, oral, 3 times/wk) is shown. When the tumor of any mouse implanted with the same original tumor reached ~3,000 mm^3^, control and treated mice implanted with the same tumor were sacrificed and examined. (B) Graph shows calculated RTVs (RTV of AZD2281-treated tumor/RTV of sham-treated tumor × 100) for tumors at 1 week after irradiation. (C) Heat map showing alterations of selected genes according the response to AZD2281 in the allograft model (*P* < 0.01). Tumor samples were sorted with respect to their RTV to highlight correlations with gene expression. (D) Overview of the allograft model and combined treatments. Eight spontaneously developed mammary tumors were collected from *Brca1^co/co^MMTV-Cre* mice and transplanted into *Balb/c-*nude mice. Corresponding tumors grown under mock conditions were compared with those treated with irradiation (10 Gy), AZD2281 (100 mg/kg, oral, 3 times/week), or their combination. After tumors reached ~3,000 mm^3^, mice were sacrificed and examined. (E) Eight RTVs comparing responses 1 week after treatment with initiation of treatment are shown. (F) Responses of allograft *Brca1*-mutant mammary tumors depend on treatment modality. Graphs show RTVs between post-treatment and baseline (start of treatment). (G) Graph indicates the time required for tumor volumes to reach 3,000 mm^3^ (**P < 0.01).

To determine whether inhibition of PARP attenuates progression of *BRCA1*-mutant breast cancer, we examined the efficacy of AZD2281 *in vivo* using the same set of allograft models as used for irradiation studies. Twelve tumor samples were orthotopically transplanted into the mammary gland of female *Balb/c-*nude mice and the effect of AZD2281 was examined by comparing vehicle-treated and AZD2281-treated tumors with the same origin (Fig. 5A). The point at which the size of the tumor in any given recipient reached ~3,000 mm^3^ was used as the endpoint for examining the drug response (i.e., RTV). The overall RTV for AZD2281-treated mice bearing *Brca1*-mutant tumor allografts was 59.5% compared with vehicle-treated controls (Fig. 5B). An analysis of tumors in individual mice treated with AZD2281 revealed that 7 of 12 mice exhibited more than a 59.5% reduction in tumor volume in response to PARP inhibition, whereas the remainder showed less than a 59.5% reduction. The less-responsive group (RTV > 59.5, n = 5) showed an 8.8% reduction in RTV following AZD2281 treatment relative to untreated tumors, whereas the more-responsive group (RTV < 59.5, n = 7) exhibited a 63.6% decrease in RTV. In an effort to identify underlying causes of the different responses, we classified tumors based on their responsiveness to AZD2281 treatment and examined gene expression patterns in the corresponding baseline tumor tissue samples. Twelve non-treated allograft mammary tumors were collected for this purpose. The whole transcriptome was screened using mRNA sequencing, and the same pipeline described above was used to identify genes whose expression correlated with RTV following AZD2281 treatment of *Brca1*-deleted tumors. A total of 674 highly correlated genes (Spearman's rank correlation > 0.6 or < -0.6, *P*-value < 0.01) were identified (Fig. 5C, and Supplementary Table 3).

To determine whether combining irradiation and PARP inhibition more effectively suppressed *BRCA1*-mutant breast cancer, we tested the efficacy of this combination in a preclinical allograft model.

Eight set of tumor-engrafted mice were divided into the following groups (n = 8/group): vehicle control; irradiation (10 Gy); AZD2281; and irradiation plus AZD2281 (Fig. 5D). Seven days after the initiation of treatment, the volumes of tumors in vehicle-treated mice had increased 9.04-fold relative to baseline, whereas tumors in mice treated with irradiation or AZD2281 were smaller, exhibiting only 5.81- and 6.04-fold increases, respectively. In mice co-treated with irradiation plus AZD2281, tumor volume was only increased 4.05-fold at this same time, indicating that these agents exerted an additive suppressive effect on tumor growth (Table 2 and Fig. 5E). We also analyzed the time-course of tumor progression for each individual after initiation of treatment by determining the time to reach a tumor volume of ~3,000 mm^3^. This analysis showed that the combination of irradiation and AZD2281 effectively delayed the progression of tumors compared with untreated mice or mice treated with irradiation or AZD2281 alone (Fig. 5F). An analysis of the time to reach a tumor volume of ~3,000 mm^3^ in individual mice treated with both X-rays and AZD2281 showed a significant 71.4% delay compared with the non-treated control group, whereas mice treated singly with X-rays or AZD2281 displayed 14.3% and 22.6% delays, respectively (Fig. 5G and Table 2).

**Figure 6:**
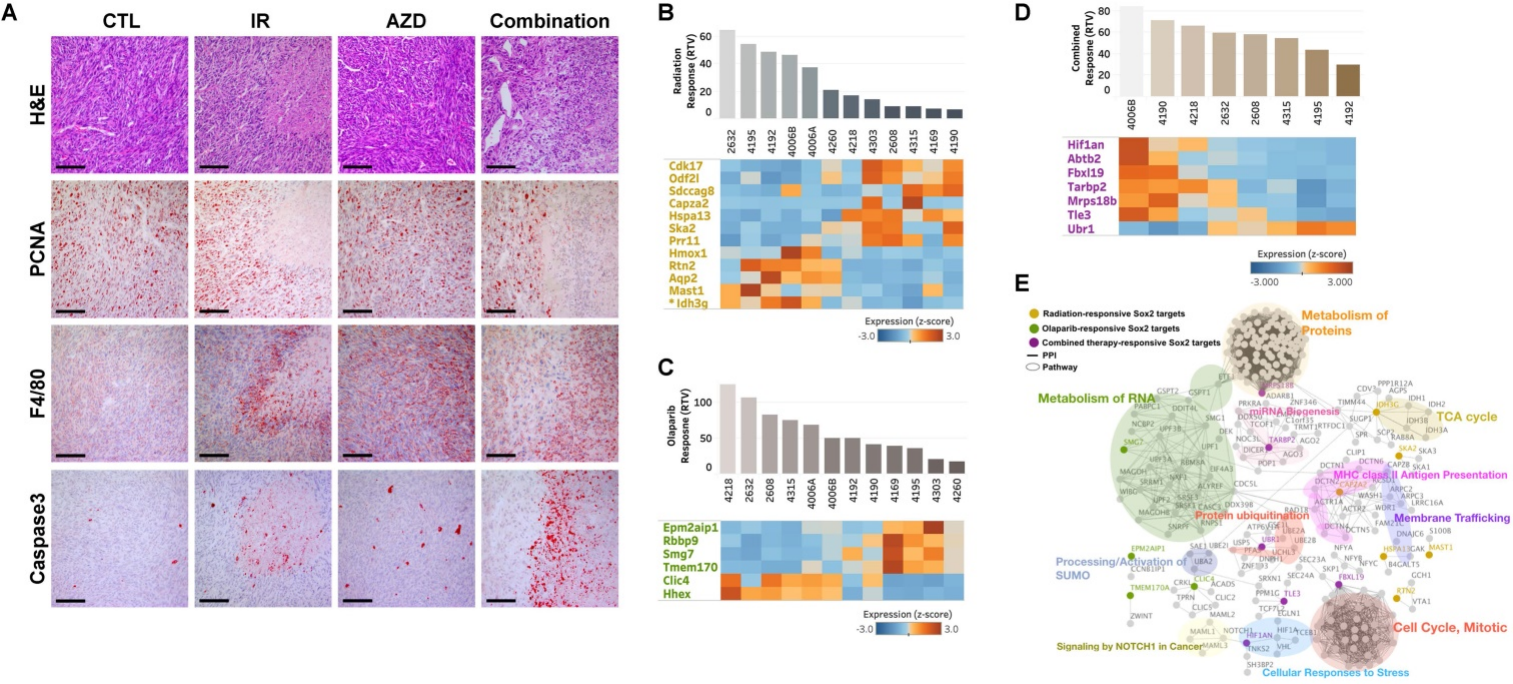
**Analysis of combined treatment response-associated biomarkers.** (A) Histological analyses of tumors from mouse 4192 treated as indicated are shown. Scale bars: 100 µm. Heat map showing down-regulation and up-regulation of selected SOX2 targets according to the response to irradiation (B), AZD2281 (C), and their combination (D) in the allograft model (*P* < 0.01). Tumor samples were sorted with respect to their RTVs to highlight correlations with gene expression. (E) Integrated functional network analysis of selected genes using the STRING protein-interaction network and KEGG pathways.

In addition, tumors from mice in the combined-treatment group displayed enlarged stromal areas with lower-intensity staining for the proliferation marker, PCNA (proliferating cell nuclear antigen), higher-intensity staining for cleaved Caspase 3, and infiltration of macrophages. Taken together, these results suggest that co-treatment with irradiation and a PARP inhibitor facilitates growth-suppression and degeneration of *BRCA1*-associated mammary tumors (Fig. 6A).

Next, we interrogated the putative drug-response marker genes in response to this combination, comparing three sets of responsive markers for irradiation, AZD2281, and their combination (Supplementary Table 1, 3, and 5). Although there were no combination-response markers in common with our previous irradiation- or AZD2281-responsive markers at the gene level, they shared enriched hallmark signatures in common at the gene-set level , implying that the three treatments uniquely affect different genes but combinatorially affect the same pathway (Supplementary Table 2, 4, and 6). One common pathway affected by all treatments was “SOX2 TARGETS”, corresponding to genes up-regulated by SOX2 transcriptional activity in human embryonic stem (ES) cells (*P*-value < 0.05) 33. Cells with activated SOX2 targets displayed ES cell-like signatures with elevated subsets of ES cell-associated transcription regulators, and cancer patients with similar patterns of gene expression showed poor survival. Our analysis also showed that the non-responder group exhibited higher expression of SOX2 targets compared with responders. For example, *Idh3g*, a SOX2 target whose expression is associated with poor survival in TCGA-BRCA patients, was frequently overexpressed in irradiation-resistant tumors (Fig. 3E and 3G). Other SOX2 targets were up- or down-regulated in the responder group with a given treatment (Fig. 6B, 6C, and 6D). To more specifically identify signaling pathways involved in the 25 SOX2 targets affected by individual therapies, we integrated all known interactions of proteins and canonical pathways with SOX2 targets in our irradiation-, AZD2281-, or combination therapy-response genes. This analysis showed that the disjointed sets of SOX2 targets were connected to each other through previously annotated protein-protein interactions (PPIs) in the constructed network (Fig. 6E and Supplementary Table 7), revealing linked signaling paths along which the three individual therapies exert effects combinatorially. It also revealed 11 clusters of proteins that were connected to various biological processes (*P*-value < 0.05). Among the 11 clusters, three were connected directly to SOX2 targets affected by AZD2281 alone, another two interacted with SOX2 targets affected by irradiation alone, and six were linked to SOX2 targets affected by combined therapy. Biological processes associated with combination therapy-response SOX2 targets, such as “Metabolism of Proteins”, “miRNA Biogenesis”, “Protein ubiquitination”, “Cellular Responses to Stress”, “Signaling by NOTCH1 in Cancer” and “Cell Cycle, Mitotic”, were potentially responsible for the crosstalk between AZD2281 and downstream irradiation effects via SOX2 targets. Among the genes in these clusters, *MRPS18B* (mitochondrial ribosomal protein S18B), *TARBP2* (TARBP2 subunit of RISC loading complex), *UBR1* (E3 ubiquitin-protein ligase), and *FBXL19* (F-Box and leucine-rich repeat protein 19) played central roles in linking the two components, providing a framework for better understanding the combined effect.

## Discussion

*BRCA1*-associated breast cancer is an extensively studied hereditary cancer that exhibits significantly greater genetic instability than sporadic breast cancers 34,35. Loss of BRCA1 is associated with defects in DNA damage repair, which causes the accumulation of errors in genetic material and eventually leads to tumorigenesis 3,36. Thus, because of these defects, it is not likely feasible to correct DNA damage in* BRCA1*-mutant tumor cells. Indeed, DNA-damaging anticancer drugs, including cisplatin and AZD2281, have been used in the treatment of *BRCA1*-associated breast cancer 37.

Herein, we show that radiotherapy is a viable approach for proactively treating the progression of *BRCA1*-associated breast cancer. Radiation therapy uses high doses of radiation to kill cancer cells and shrink tumors. At high doses, cancer cells whose DNA is damaged beyond repair, grow slowly and stop dividing. When the damaged cancer cells die, they are broken down, causing the tumor mass to shrink. Because the breast can be treated with minimal damage to other parts of the body, it is a suitable organ for local radiotherapy treatment. Although resection is the primary treatment option for breast cancer, radiation therapy can also be used in cases where breast tumors cannot be surgically removed or in inflammatory breast cancer, the latter of which is an aggressive type of cancer that spreads to lymph channels of the skin covering the breast 38,39.

Mutations in *BRCA1* are known to confer defects in DNA repair and increase sensitivity to DNA damage, suggesting that a lower dose of irradiation might be needed to kill *BRCA1* mutant tumor cells. Our studies using allograft mice bearing *Brca1*-mutant tumors showed that tumor growth was significantly delayed in mice treated by monotherapy with 20 Gy irradiation compared with untreated mice (RTV = 27.9%) in association with elevated levels of cleaved Caspase 3 and increased macrophage infiltration (Fig. 2B and 2E). In addition, tumors in 10 of 12 mice treated with irradiation were more than 50% smaller than those of their control counterparts (95% CI, 15.29-40.59), and none of the tumors in this cohort was larger than those of their control counterparts. Importantly, our *in vivo* results show that tumor-bearing mice harboring the same *Brca1* mutation (*Brca1-Δ11*) were not uniform in their responses to irradiation. Although X-ray treatment triggered the accumulation of DNA damage in all tested tumors (N = 12; RTV = 2.39; 95% CI, 0.38-4.39), tumor progression in some mice resembled that observed in the corresponding untreated control mice (N = 12; RTV = 7.46; 95% CI, 4.49-10.43) (Fig. 2C and Table 1). Classification of irradiated mice based on whether their RTVs were above average (non-responders) or below average (responders) revealed that the average RTVs of irradiated non-responders (N = 5) and responders (N = 7) were 4.41 (95% CI, 1.45-7.36) and 0.94 (95% CI, -1.56-3.44), respectively.

Precision medicine, also called personalized medicine, in which an individual's drug response is predicted based on an analyses of their baseline tumor, is the next horizon of efficient tumor therapy and patient safety. Although we do not yet understand the mechanisms underlying the variability in responses to a given treatment, recent findings have suggested that biomarkers can be closely correlated with sensitivity to a specific drug. Western blot analyses revealed that phospho-ERα (Ser118), phospho-CHK2 (Thr68), and p53 were frequently increased in the responder group in unirradiated baseline tumors. Interestingly, phosphorylation of p53 at serine 15, which occurs after DNA damage and reduces p53 interactions with MDM2 40, was detected in the same sets of irradiated tumors, suggesting that p53-regulatory mechanisms were maintained in these tumors (Fig. 3C). Of particular interest is our observation that, although *BRCA1*-associated mammary tumors are frequently associated with inactivation of ERα and p53, expression of these proteins was readily detected in *BRCA1*-mutant mammary tumors 41,42. Although *BRCA1*-associated mammary tumors are known as triple-negative breast cancers, histopathological analysis of *BRCA1*-associated breast cancer showed that 32.7% and 22.5% of breast cancer from *BRCA1* carrier were not triple-negative and ERα-positive, respectively. In addition, ERα-positive *BRCA1*-associated breast cancer tends to develop in older ages (>50 years) and is less aggressive than ERα-negative, implying the association of ERα status and tumor progression in *BRCA1*-associated breast cancer 42.

In the contrary, non-responder frequently showed the high level of phospho-Rb (Ser807/811) in western blot and tissue staining, and also displayed the intense EpCAM in the tumor cells which were not altered after irradiation (Fig. 3B, and 3D). It has been reported that EpCAM is involved in the response to chemotherapy/radiotherapy in a xenograft model of prostate cancer and that its knockdown causes significant tumor growth inhibition and induction of sensitivity to chemotherapy/radiotherapy 43. However, knocking down EpCAM failed to significantly alter radiation responses in *Brca1*-mutant tumor cells (data not shown), suggesting that, although EpCAM is a functional biomarker candidate, it not a promising target of therapeutics. In addition, we compared gene expression profiles of baseline tumors with the responsiveness of these tumors to X-ray treatment. This analysis identified 158 genes as radiation response-associated genes, and a further analysis of PPI networks of these genes and their interacting proteins revealed that a number of protein clusters are associated with radiation responsiveness. These include the breast cancer progenitors, *SEMA5A, KITL, CAV2, EPS8* and *PKP4*, and the Rb1-targeted senescence-associated proteins, *FRMD4A, SKA2, PRR11* and *ITGBL1*. Additionally, these genes encode proteins that are also involved in biological adhesion, cellular proliferation, cytoskeleton function, locomotion, movement of the cell or a subcellular component, or protein modification processes.

AZD2281, an inhibitor of PARP, which senses DNA strand breaks and is essential for various forms of DNA repair, has demonstrated radiosensitization in multiple cancers 32,44,45. In *BRCA1*-associated breast cancer, a clinical trial showed that progression-free survival at a median follow-up of 14 months was 2.8 months longer and the risk for disease progression or death was 42% lower with AZD2281 monotherapy compared with conventional chemotherapy 9. Thus, we tested the efficacy of concurrent treatment with AZD2281 and irradiation radiotherapy in controlling tumor growth and overall survival in allograft mice bearing *Brca1*-mutant tumors compared with AZD2281 or radiotherapy alone. Our *in vivo* studies showed that tumor growth was significantly delayed in mice treated with combined irradiation (10 Gy) and AZD2281 (100 mg/kg) (RTV = 4.05) compared with untreated mice (RTV = 9.04). This delay is further reflected in the time required for tumors to reach a volume of 3,000 mm^3^, which was significantly longer for mice receiving combined treatment (mean, 28.8 days; 95% CI, 22.89-34.61) than for untreated mice (mean, 16.8 days; 95% CI, 12.74-20.76). By comparison, both monotherapies failed to produce significant differences in tumor growth or survival compared with the untreated condition (Fig. 6 and Table 2). These results are also in accord with our histological analysis showing a reduction in proliferation (PCNA), induction of apoptosis (cleaved Caspase 3), and infiltration of immune cells (Fig. 6E). Taken together, our results provide evidence that combined treatment offers the prospect of broadening the clinical benefit of AZD2281 beyond its use as a monotherapy.

Treatment options for *BRCA1*-associated breast cancers have been limited by many clinical trial-related hurdles. Thus, simulating the clinical situation using a mouse model, such as the *Brca1*-mutant tumor-bearing mouse model, is a useful strategy for testing treatment efficacy. Results of the current study based on this preclinical system suggest that patient-specific radiation therapy and AZD2281 concurrent treatment could be a useful strategy for controlling *BRCA1*-associated breast cancer. Additional studies are needed to confirm the potential of this strategy and the utility of these markers for future clinical applications of irradiation responsiveness in *BRCA1*-associated breast cancer.

## Supplementary Material

Supplementary table S1.

## Figures and Tables

**Table 1:** Summary of results of a preclinical experiment testing irradiation effects on the growth of *Brca1*-associated mammary tumors

	Treatment	RTV^1^	*P* value
Total	Control (N = 12)	7.46 ± 6.16	
	Irradiation (N = 12)	2.39 ± 3.35	0.054
Non-responder	Control (N = 5)	9.32 ± 9.53	
	Irradiation (N = 5)	4.41 ± 4.68	0.650
Responder	Control (N = 7)	6.12 ± 1.89	
	Irradiation (N = 7)	0.94 ± 0.34	0.001

^1^RTV, ratio of tumor volume 1 week after treatment versus baseline (at inception of treatment).

**Table 2:** Summary of results of a preclinical experiment testing the combined effects of irradiation and AZD2281 treatment on *Brca1*-mutant tumors

	Treatment	Control	Irradiation	AZD2281	Combination
RTV^1^	Mean	9.04	5.81	6.08	4.05
	SD	5.64	3.35	2.62	2.98
	95% CI	4.318-13.75	3.01-8.60	3.90-8.27	1.56-6.54
	*P*	-	0.102	0.156	0.028
Survival	Mean	16.8	19.2	20.6	28.8
	SD	5.12	5.44	8.16	9.65
	95% CI	12.74-20.76	15.24-23.26	15.46-25.79	22.89-34.61
	*P*	-	0.36	0.27	0.008

^1^RTV, ratio of tumor volume 1 week after treatment versus baseline (at inception of treatment).
